# Rethinking Pathways: Innovative Approaches to Identify Individuals Experiencing First-Episode Psychosis and Connect Them to Care

**DOI:** 10.1192/j.eurpsy.2025.502

**Published:** 2025-08-26

**Authors:** P. Mohr, P. Falkai, G. Dom, P. Boyer, S. Galderisi, B. Chaumette, W. Gaebel, C. Arango, S. Dolffus, I. Bitter, P. Keri, A. Vita, C. Duarte, A. Catalan, P. Toczyski, J. Beezhold, J. Samochowiec, J. Rybakowski, A. Szulc, M. Nordentoft, O. Howes, P. Fusar-Poli, J. Vasco Santos, F. Nolan, V. Quoidbach

**Affiliations:** 1 National Institute of Mental Health, Prague, Czech Republic; 2 Department of Psychiatry and Psychotherapy, Munich University Hospital, Munich, Germany; 3 Collaborative Antwerp Psychiatric Research Institute, University of Antwerp, Antwerp; 4 European Brain Foundation, Brussels, Belgium; 5 Department of Mental and Physical Health and Preventive Medicine, University of Campania “Luigi Vanvitelli”, Naples, Italy; 6 Université Paris Cité, Institute of Psychoatry and Neuroscience of Paris (IPNP), INSERM U1266, GHU-Paris Psychiatrie et Neursociences, Hôpital Sainte Anne, Paris, France; 7 LVR-Klinikum, Department of Psychiatry and Psychotherapy, Medical Faculty, Heinrich-Heine-University, Dusseldorf, Germany; 8 Child and Adolescent Department, Institute of Psychiatry and Mental Health, Hospital General Universitario Gregorio Marañón, CIBERSAM, IiSGM, School of Medicine, Universidad Complutense, Madrid, Spain; 9 University Caen Normandie, Caen, France; 10 Department of Psychiatry and Psychotherapy, Semmelweis University, Budapest, Hungary; 11 GAMIAN-Europe , Brussels, Belgium; 12 University of Brescia, School of Medicine, Brescia, Italy; 13 European Federation of Psychologists’ Associations, Standing Committee on Clinical Neuropsychology, Brussels, Belgium; 14 Department of Neuroscience, University of the Basque Country UPV/EHU, Bilbao, Spain; 15 Maria Grzegorzewska University (APS), Warsaw, Poland; 16 Great Yarmouth Acute Service, Norfolk and Suffolk NHS Foundation Trust, Northgate Hospital, Great Yarmouth, United Kingdom; 17 Department of Psychiatry, Pomeranian Medical University, Szczecin; 18 Department of Adult Psychiatry, Poznan University of Medical Sciences, Poznan; 19 Medical University of Warsaw. Department of Psychiatry, Warsaw, Poland; 20 Department of Clinical Medicine, University of Copenhagen, Copenhagen, Denmark; 21 Institute of Psychiatry, Psychology and Neuroscience, King’s College London; 22 Department of Psychosis Studies, Institute of Psychiatry, Psychology and Neuroscience, King’s College London , London, United Kingdom; 23 Department of Community Medicine, Information and Health Decision Sciences, Faculty of Medicine, University of Porto, Porto, Portugal; 24 Mental Health Nursing Anglia Ruskin University, Cambridge, United Kingdom; 25 European Brain Council , Brussels, Belgium

## Abstract

**Introduction:**

Psychotic disorders, particularly schizophrenia, are severe mental illnesses associated with high rates of disability and functional impairment, causing significant individual burden and incurring high societal costs. Typical onset of schizophrenia is in late adolescence or early adulthood and the complex management requires often life-long pharmacological and non-pharmacological treatment. Early symptom recognition and timely intervention can improve the course of illness and result in better outcome and prognosis, effective management leads to a functional recovery. However, recent reports have identified significant gaps in access to timely assessment and shared decision-making interventions, with inadequate care pathways. In the face of an unprecedented demand for mental healthcare for young people, it can be challenging for health services to deliver high-quality mental healthcare which, according to the World Health Organization, should be timely, effective and evidence-based, safe and person-centered. The project covers nine countries in Europe.

**Objectives:**

Building on the European Brain Council Rethinking Schizophrenia Beyond The Voices Policy Report (2024), the survey and literature review aim to: (1) evaluate the effectiveness of integrated models of youth mental healthcare on a broader range of outcomes, including both mental health outcomes, such as clinical symptoms, functioning and quality of life and health service outcomes, including access and satisfaction with care in young people; and (2) identify the common components of integrated care pathways for young people with first episode psychosis.

**Methods:**

Using the care pathway as a tool at the first step of the research, a cross-country survey was co-designed with the Board of experts and anonymously launched earlier this year. By complementing the survey, the literature review on the care pathway will address quality and continuity of care from the first onset of psychosis and schizophrenia to long-term care in the selected countries including existing guidelines and overview country health situation assessments.

**Results:**

Patients and mental health professionals’ insights will be collected. Obtained data will also be analysed by the stakeholders and used to formulate recommendations for policy makers, care payers, mental health professionals, patients and their families (both country specific and at the EU level).

**Conclusions:**

A policy report, based on the consensus, will be released at the Brain Awareness Week 2025 with results and recommendations which will provide valuable insight into understanding the needs of patients with first-episode psychosis and defining the optimal care pathways to engage with them. In order to show that there is a progress in the field of care for schizophrenia patients, the utilization of new technologies is included.

**Disclosure of Interest:**

None Declared

